# Transcriptome analysis reveals important candidate genes involved in grain-size formation at the stage of grain enlargement in common wheat cultivar “Bainong 4199”

**DOI:** 10.1371/journal.pone.0214149

**Published:** 2019-03-25

**Authors:** Yuanyuan Guan, Gan Li, Zongli Chu, Zhengang Ru, Xiaoling Jiang, Zhaopu Wen, Guang Zhang, Yuquan Wang, Yang Zhang, Wenhui Wei

**Affiliations:** 1 College of Life Science and Technology, Henan Institute of Science and Technology / Collaborative Innovation Center of Modern Biological Breeding, Henan Province, Xinxiang, China; 2 Xinyang Agriculture and Forestry University, Xinyang, China; Murdoch University, AUSTRALIA

## Abstract

Grain-size is one of the yield components, and the first 14 days after pollination (DAP) is a crucial stage for wheat grain-size formation. To understand the mechanism of grain-size formation at the whole gene expression level and to identify the candidate genes related to grain pattern formation, cDNA libraries from immature grains of 5 DAP and 14 DAP were constructed. According to transcriptome analysis, a total of 12,555 new genes and 9,358 differentially expressed genes (DEGs) were obtained. In DEGs, 2,876, 3,357 and 3,125 genes were located on A, B and D subgenome respectively. 9,937 (79.15%) new genes and 9,059 (96.80%) DEGs were successfully annotated. For DEGs, 4,453 were up-regulated and 4,905 were down-regulated at 14 DAP. The Gene Ontology (GO) database indicated that most of the grain-size-related genes were in the same cluster. The Kyoto Encyclopedia of Genes and Genomes (KEGG) database analysis showed that 130, 129 and 20 DEGs were respectively involved in starch and sucrose metabolism, plant hormone signal transduction and ubiquitin-mediated proteolysis. Expression levels of 8 randomly selected genes were confirmed by qRT-PCR, which was consistent with the transcriptome data. The present database will help us understand the molecular mechanisms underlying early grain development and provide the foundation for increasing grain-size and yield in wheat breeding programs.

## Introduction

Wheat is the second largest crop in the world, it feeds nearly 30% of the world population [1]. With a growing human population and rising demand for wheat, there is a great challenge to global wheat security. Therefore, there is an urgent need to increase wheat yield and improving grain yield is becoming the major goal of wheat breeding. Grain size and weight are major determinants of wheat yield [2], which are also found to be associated with wheat flour quality [3]. Thus, understanding the regulatory mechanisms underlying early gene expression and selecting the appropriate candidate genes for grain size/weight are of great significance for yield and quality improvement in wheat.

The major way to explore the mechanisms of grain development is to identify gene activities and functions. Many grain-size/weight genes have been successfully cloned in plants including rice and *Arabidopsis thaliana* [4–6]. More recently, studies show that LTBSG1 regulates panicle and grain development in rice by brassinosteroid biosynthetic pathway [7]. *OsGW2*, encoding a ring-type E3 ubiquitin ligase, affects grain width, weight and yield by regulating cell division [8]. *OsGW5* regulates grain development through the ubiquitin-proteasome pathway [9]. In *A*. *thaliana*, many genes were found to be involved in regulating grain development. *TTG2* and *AP2* promote grain growth by increasing cell expansion in the integuments [10]. The *ARF2* gene, involved in auxin signaling, regulates grains size by controlling cell division [11]. Genes regulating grain development, mainly involved in hormone pathway and ubiquitin-proteasome pathway have been identified in model plants in previous studies which provides useful information for this study.

Classical investigations into molecular genetic bases of grain development in higher plants have been conducted mainly through map-based cloning. However, it is time-consuming to clone genes involved in wheat grain-size/weight by map-based cloning because of the large and complex genome. Therefore, sometimes the functional genes related to grain-size/weight are screened by their orthologous genes in rice and other species. *TaGS-D1* and *TaCYP78A5*, associated with grain-size/weight were cloned by using probes from rice *OsGS3* and Arabidopsis *CYP78A5* to search wheat expressed sequence tag (EST) database in GenBank [12–13]. Cytokinin oxidase/dehydrogenase (CKX) plays an important role in plant development and wheat *TaCKX6a* is identified to function in grain-size/weight and filling rate by comparing different recombinant inbred lines (RIL) [14]. In addition grain-size/weight is a complex agronomic trait controlled by quantitative trait loci (QTL) [15] and some QTLs related to wheat grain-size/weight have been identified [16]. Association analysis revealed that wheat TaGW2, an E3 ubiquitin ligase, affects grain weight and width by controlling endosperm cell number [17]. Although several genes regulating wheat grain size and weight have been cloned, it is far from investigating the mechanisms of grain development and needed to identify more candidate genes for grain-size/weight to understand wheat seed development.

Global transcriptome analysis is becoming a feasible and effective method to analyze genes expression profiles in different spatial or temporal samples and identify important genes. Based on abundant annotated gene database, many studies on grain development have been performed in several plant species by analyzing dynamic genes expression during grain development. In *Brassica napus*, genes critical for embryo development were identified to be closely linked to cell division, lipid and protein metabolisms and signal transduction [18–19]. Gene expression profiles of developing soybean seeds revealed that genes involved in grain development are related to oil and protein biosynthesis, energy metabolism and signal transduction [20–22]. A transcriptomic study revealed that the expression levels of many transcription factors and grain-specific genes changed significantly during maize grain development [23]. In addition, transcriptome analysis has also been used to study grain development in foxnut and peacan, and many candidate genes have been identified [24–25]. Therefore, transcriptomic data is not only an alternation of exploring grain development-related genes but it also serves as a valuable resource for further understanding the key gene regulatory mechanisms during plant seed development.

On the basis of growth phases, grain development has been divided into three phases: grain enlargement (0–14 DPA), grain filling (15–35 DPA) and physiological maturity (36–50 DPA) [26]. Grain enlargement is the first stage of grain development, in which the basic morphogenesis of grain is established and grain filling is initiated. Grain enlargement stage is very important during the whole grain development and determine grain-size and affect next grain-weight. Considering the importance of grain enlargement phase, it is indispensable to reveal the specific events that take place at this phase. There is little reports on the genetic regulation mechanism of wheat grain enlargement, except for several reports that described genetic events at this stage. According to previous studies, earliest grain development period (6–10 DAP) is strongly associated with cell division, photosynthesis and development rather than storage product synthesis, while transcription factors were involved in early grain development [27]. In this study, transcriptomes of wheat grains at 5 and 14 days after pollination (DAP) were analyzed to examine their gene expression profiles and to monitor changes of gene expression. Our study aims to analyse key genes that may be related to grain enlargement and provide theoretical help for high-yield wheat breeding.

## Results

### Sequence assembly based on cDNA libraries sequencing data

By means of construction and sequencing of two cDNA libraries, a total of 4.73 G and 3.88 G nucleotides were generated from T05 (5 DAP) and T06 (14 DAP) libraries. The sequencing raw data have been submitted to NCBI and the accession number is PRJNA524238. N (unread bases) content was 0% in both libraries. GC content, Q20 and Q30 is 54.85%, 92.07% and 85.96% for T05, and 53.76%, 91.43% and 84.97% for T06, respectively (Table 1). Q20 and Q30 refer to a 1% chance of error and 99% confidence and a 0.1% chance of error and 99.9% confidence, respectively. These results indicate that all the data were qualified for downstream analysis.

**Table 1:** Summary of sequencing outcomes from grain enlargement stage.

Sample	ReadSum(Gb)	GC (%)	N (%)	Q20 (%)^a^	Cycle Q20 (%)	Q30 (%)^b^
T05	4.73	54.85	0	92.07	100	85.96
T06	3.88	53.76	0	91.43	100	84.97

N% indicates the ratio of unread bases in the total base number.

^a^ Q20 indicates a quality score of 20, a 1% chance of error and 99% confidence

^b^ Q30 indicates a quality score of 30, a 0.1% chance of error and 99.9% confidence

### Identification of wheat new genes and differential expression of genes (DEGs)

A total of 112,984 genes were generated from two cDNA library. After filtering those genes that contained only one exon or encoded short peptide chains, a total of 12,555 new genes were revealed by blasting the reference genome using Cufflinks. The detailed information and sequences are listed in S1 Table.

**Fig 1:**
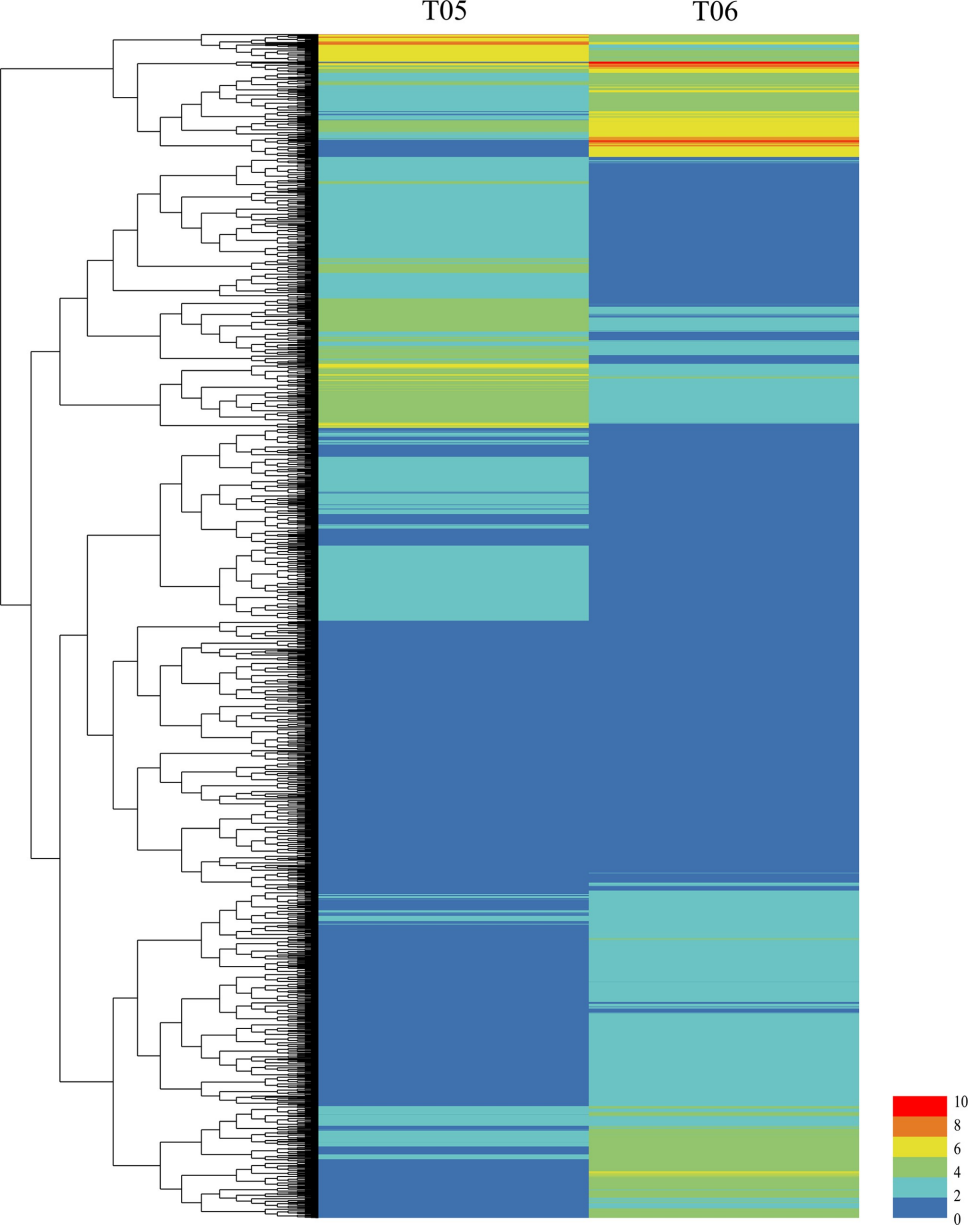
Hierarchical clustering analysis of DEGs between T05 and T06. T05, 5 DAP; T06, 14 DAP. A total of 9,358 genes were identified at two developmental stages. The color key represents the FPKM (Fragments Per Kilobase of exon per Million fragments mapped) normalized log2 transformed counts. Red and blue represent high and low expression. Each row represents a gene.

**Fig 2:**
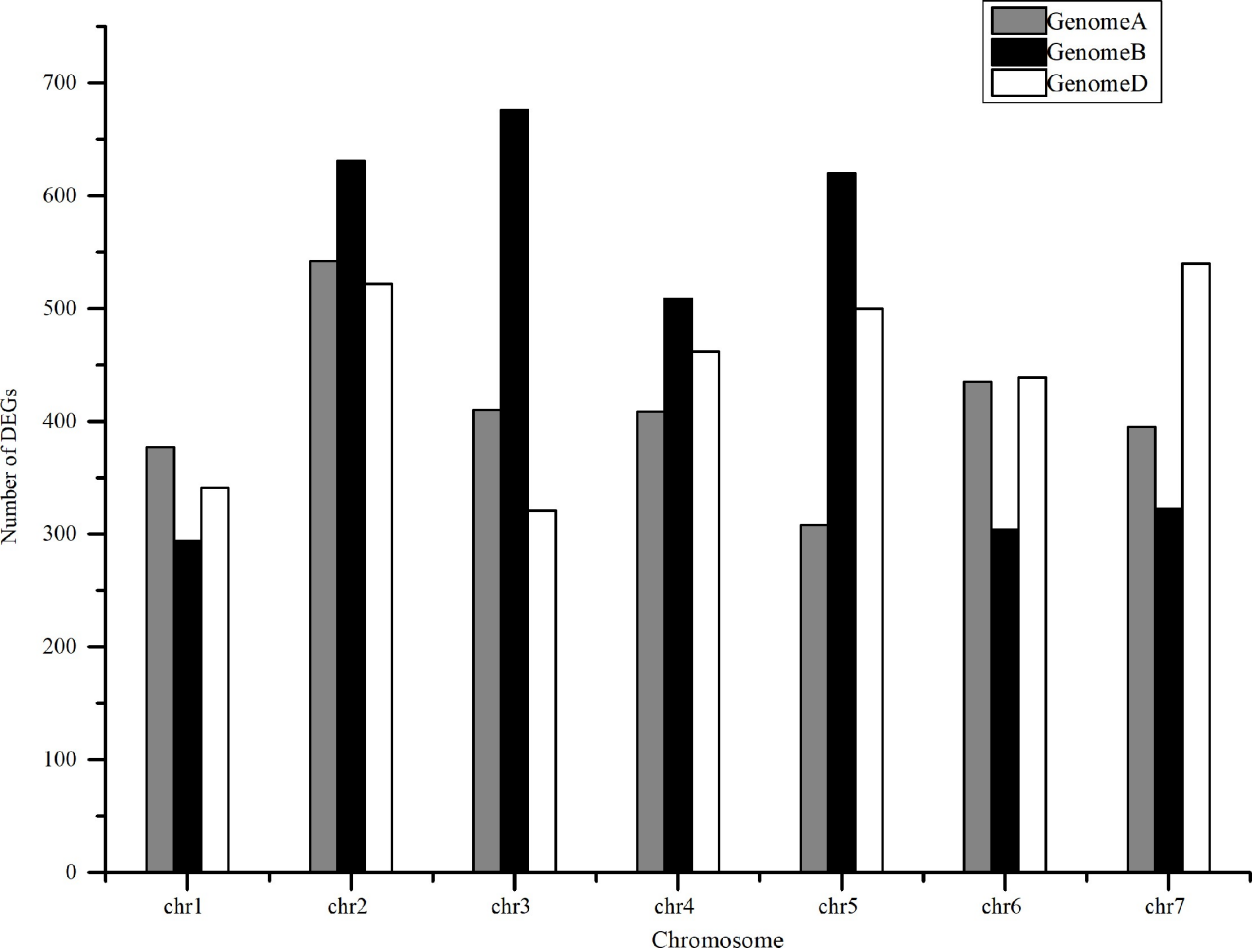
Number of DEGs located at different chromosome on subgenome A, B and D.

To identify the DEGs between grains of 5 DAP and 14 DAP, FPKM of each gene was calculated. There are 9,358 DEGs in total (S2 Table), and DEGs were shown by heat map (Fig 1). Among these DEGs, 2,876, 3,357 and 3,125 DEGs were located to A, B and D genome respectively. The most DEGs (676) were located on chromosome 3B (Fig 2).

### Functional analysis of DEGs

**Fig 3:**
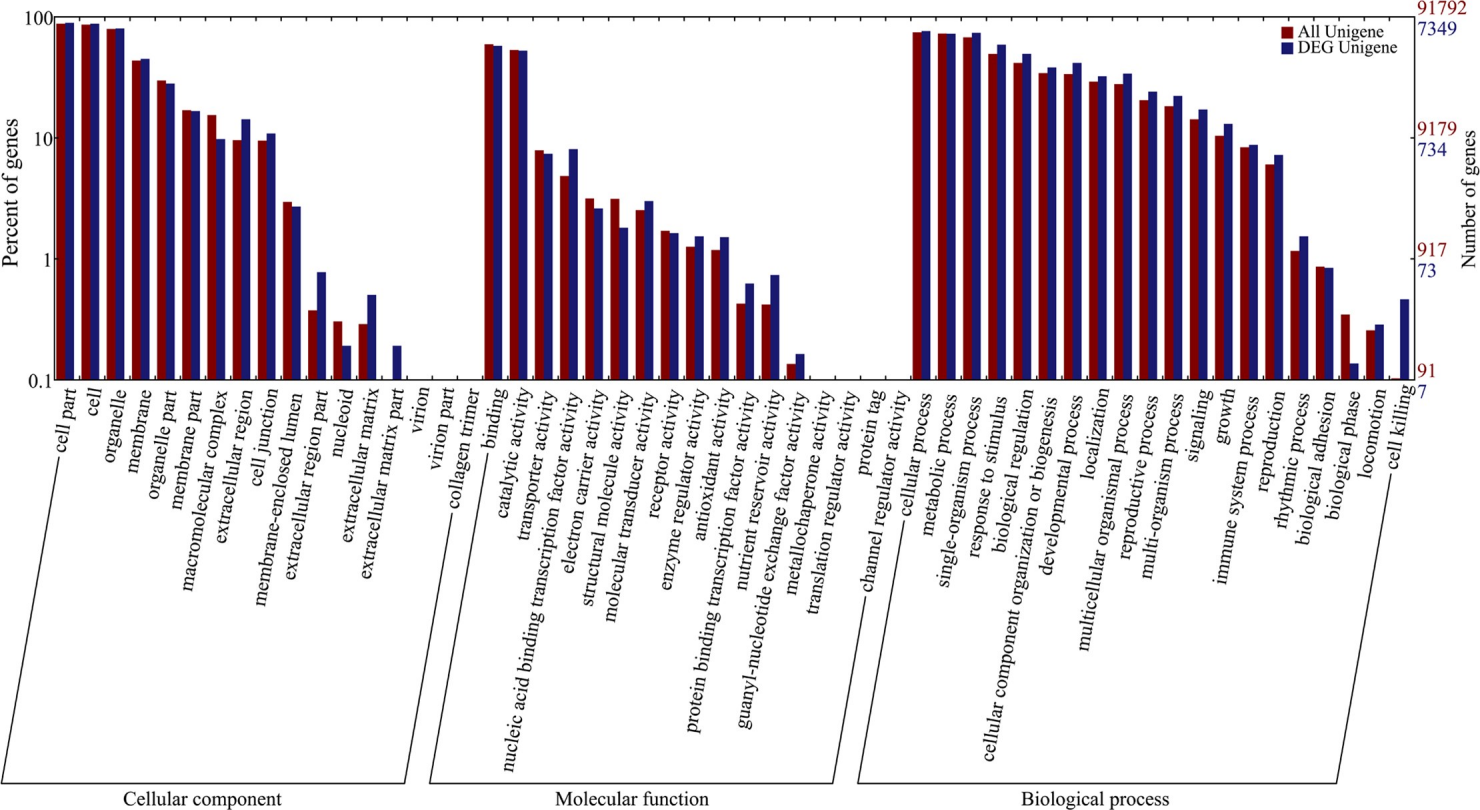
Histogram of Gene Ontology (GO) classifications of DEGs between 5 DAP and 14 DAP. A total of 7,349 DEGs were assigned to three main GO functional categories and then divided into 54 sub-categories.

To analyze functions of DEGs, above 7,349 DEGs were used by GO analysis. These DEGs are classified into 54 functional groups in three main categories (cellular component, molecular function and biological process) (Fig 3, S3 Table). GO annotation results showed that the terms of cell part, cell and organelle were dominant in the cellular component, those of binding and catalytic activity were primary in molecular function, and those of metabolic process, cellular process and single-organism process were foremost in biological process (Fig 3). These GO terms demonstrated that genes related to wheat early grain development encoded diverse regulators and protein.

**Fig 4:**
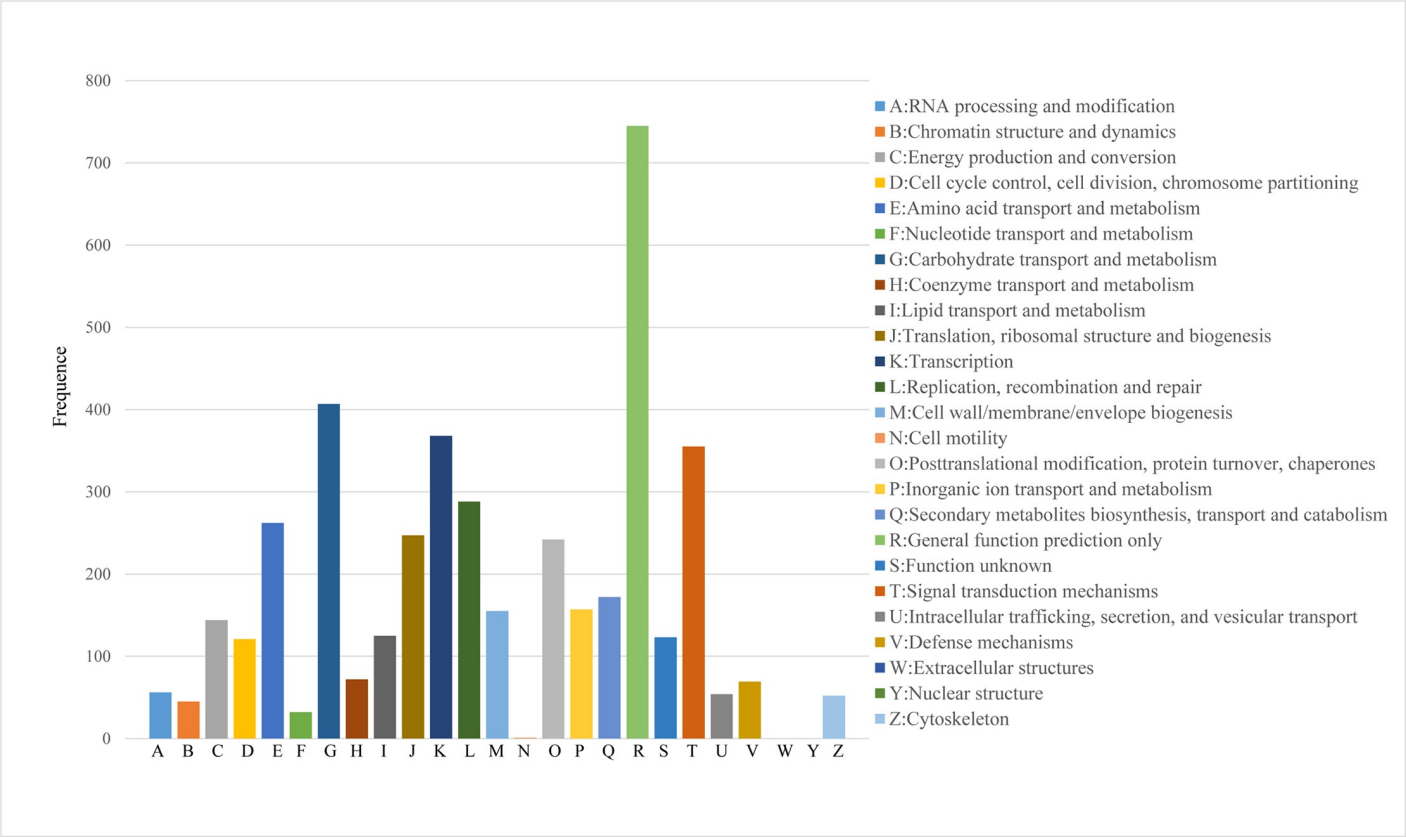
Clusters of orthologous groups (COG) function classification of DEGs between 5 DAP and 14 DAP. A total of 2,968 DEGs were distributed across 25 COG categories.

The COG functional classification showed that 2,968 DEGs were distributed across 25 COG categories (Fig 4, S4 Table). It also showed that General function prediction only accounts for the largest percentage and followed by carbohydrate transport and metabolism. Many DEGs were also involved in cluster transcription and signal transduction mechanism. These results indicated that genes in above categories play important roles in the early grain development.

**Fig 5:**
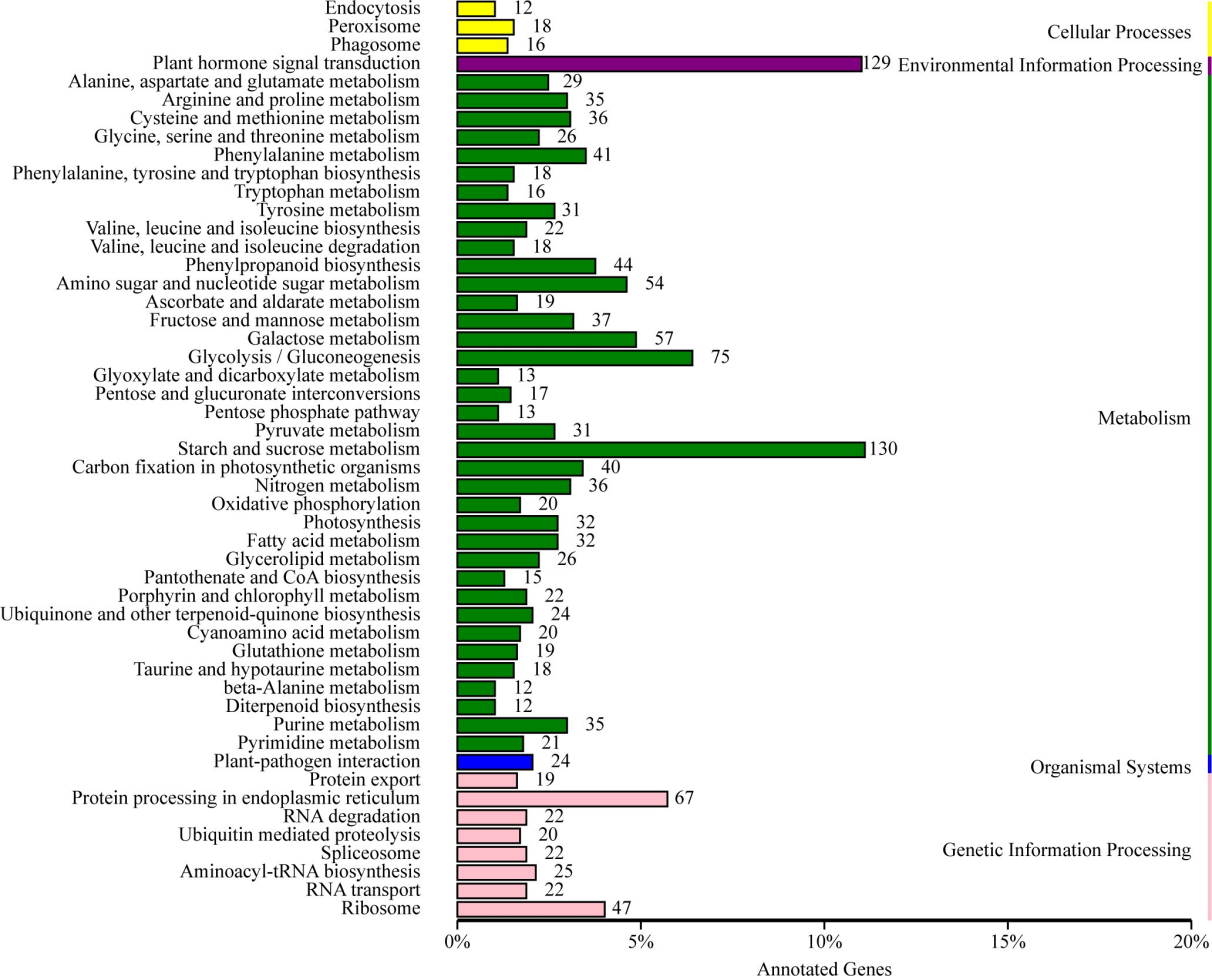
Clusters of KEGG function classification of DEGs. A total of 1,920 DEGs were mapped to 107 KEGG pathways.

To further understand the physiological processes of grain development after flowering, the KOBAS software was used to test the statistical enrichment of DEGs in KEGG pathways and it was found that there were 1,920 DEGs mapped to 107 KEGG pathways (S5 Table). Most of the correlative genes were differently expressed for ubiquinone, other terpenoid-quinone biosynthesis, phenylalanine metabolism and tyrosine metabolism. Among the 107 pathways, the largest pathway was starch and sucrose metabolism (130 genes), followed by plant hormone signal transduction (129 genes), glycolysis/gluconeogenesis (75 genes) and protein processing in endoplasmic reticulum (67 genes) (Fig 5). Starch is the major component of wheat grains, which is considered as a key determinant of wheat yield. The results of this study showed that 130 DEGs were involved in starch and sucrose metabolism in early grain formation, indicating that these genes expressed at 14 DAP much more likely play important roles for following grain-filling developmental stage.

Additionally, 9,937 (79.15%) new genes and 9059 (96.80%) DEGs were successfully annotated using the NCBI's NR database and the Swiss-Prot database respectively (Table 2).

**Table 2:** Functional annotation of new genes and differentially expressed genes in the COG, GO, KEGG, Swiss-Prot and NR databases.

Annotation Database	Number of Annotated New Genes	300< = Length<1000	Length> = 1000	Number of Annotated DEGs
**COG_Annotation**	640 (5.1%)	346	271	2,968 (31.72%)
**GO_Annotation**	3,626 (28.88%)	1871	1630	7,349 (78.53%)
**KEGG_Annotation**	784 (6.24%)	447	298	1,669 (17.84%)
**Swissprot_Annotation**	3,919 (31.21%)	1843	1977	6,786 (72.52%)
**NR_Annotation**	9,887 (78.75%)	5554	3994	9,055 (96.76%)
**All_Annotated**	9,937 (79.15%)	5585	4013	9,059 (96.80)

### Identification of candidate genes related to early grain development

In the present study, phytohormones play an important role in seed development, this was reflected by transcripts for the majority of genes related to synthesis and signaling of phytohormones. One hundred twenty-nine hormone-related DEGs were significantly changed from 5 DAP to 14 DAP (Fig 5). The results showed that the expression of 17 DEGs (4 up-regulated and 13 down-regulated) related to auxin signal significantly changed. The expression of *DELLA* (K14494 Traes_4BS_2EE4988CD), which is a suppressor of the GA signaling pathway, significantly decreased. KEGG pathway for the hormone signal transduction was shown in S1 Fig.

DEGs were almost mapped to sucrose and starch metabolic pathway in KEGG, and most of DEGs were up-regulated, such as sucrose-phosphate synthase (EC2.4.1.14) which was over 2 folds changed; trehalose 6-phosphate synthase (EC2.4.1.15) was over 2 folds changed; sucrose synthase (EC2.4.1.13) was over 3 folds changed; trehalase (EC3.2.1.28) was over 2.5 folds changed (S3 Fig). The phenylalanine metabolism was also enriched, and 41 DEGs related to this pathway significantly changed. Among 41 DEGs, 19 and 22 DEGs were up and down-regulated (S3 Fig).

**Fig 6:**
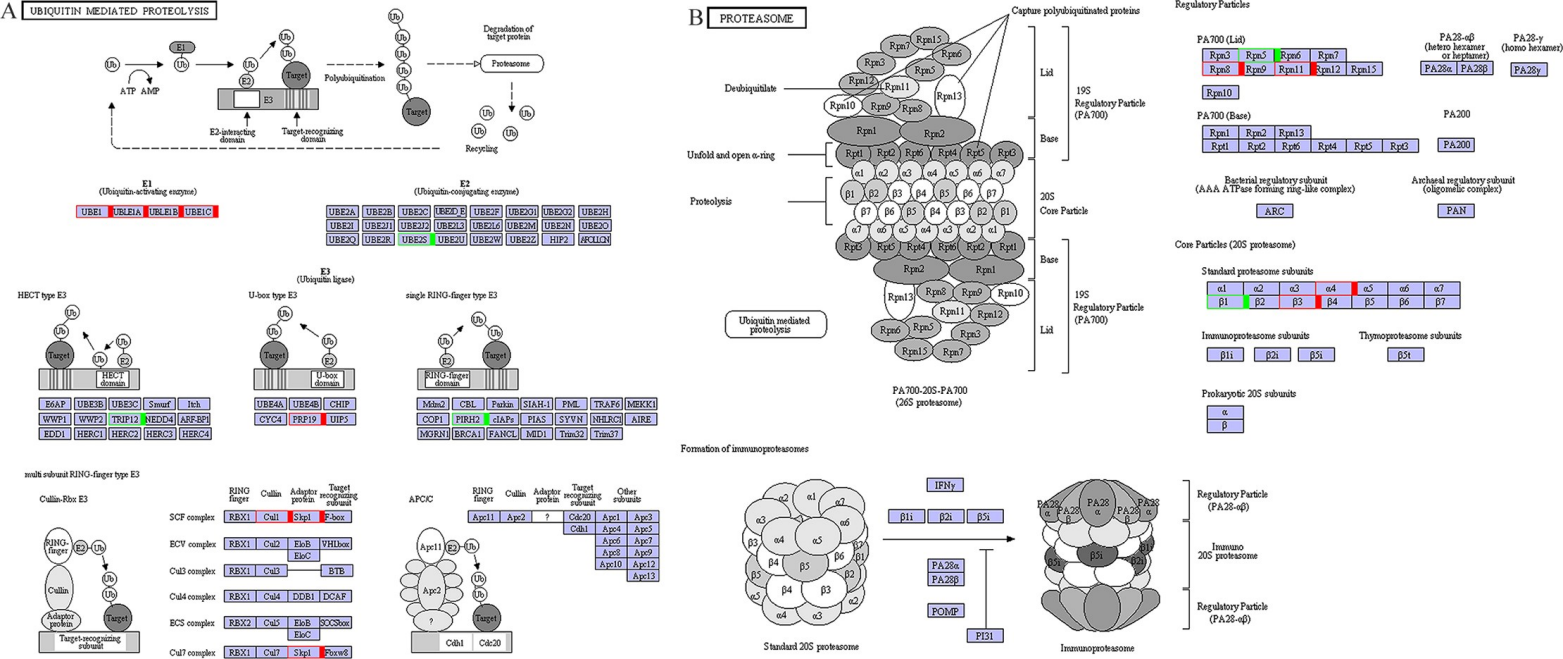
KEGG pathway analysis identified the ubiquitin-mediated proteolysis pathway (ko04120) and proteasome pathway (ko03050) for DEGs between 5 DAP and 14DAP. Red, green and blue indicate genes expression increase, decrease and mixed change at 14 DAP, respectively. (A) ko04120, and (B) ko03050.

Ubiquitin-proteasome system is one of the most important mechanisms implicated in grain developmental processes. KEGG pathway analysis identified 20 and 6 DEGs for the ubiquitin-mediated proteolysis pathway (ko04120) (Fig 6A) and proteasome pathway (ko03050) (Fig 6B) (Table 3).

**Table 3:** Summary of DEGs related to grain size/weight (ubiquitin-mediated proteolysis pathway and proteasome pathway) in the KEGG database between T05 and T06.

Pathway type	Pathway ID	DEG number	Up/Down (numbers of genes)	Gene ID
Ubiquitin-mediated proteolysis	ko04120	20	17(Up)	Traes_1BS_3644A9277; Traes_7AL_7DB8A9E73 Traes_1DL_33A487FF6; Traes_7AL_4F3563535 Traes_1DS_2180FD022; Traes_6BS_60289FA38 Traes_2BS_1D691A35F; Traes_5DS_3276C3054 Traes_2BS_20BCFE1EE; Traes_5BS_CCDBFFE79 Traes_3B_852298DCF; Traes_5AS_8A0E6E7AD Traes_4AL_F741E66BB; Traes_4DL_99F095583 Traes_4AS_091CDD198; Traes_4DL_9992A99F2 Traes_4DL_99308AE34
			3(Down)	Traes_1AL_4820D80E2; Traes_6BS_0A38324FC Traes_1DL_F58BB4E2B
Proteasome	ko03050	6	4 (Up)	Traes_2BL_1D52B3866; Traes_7BS_E2E7D3A0B Traes_4DS_5682875B0; Traes_7DL_9BA1451D6
			2(Down)	Traes_1BL_CF0FA226A; Wheat_newGene_6789

### Gene expression level validation by qRT-PCR

**Fig 7:**
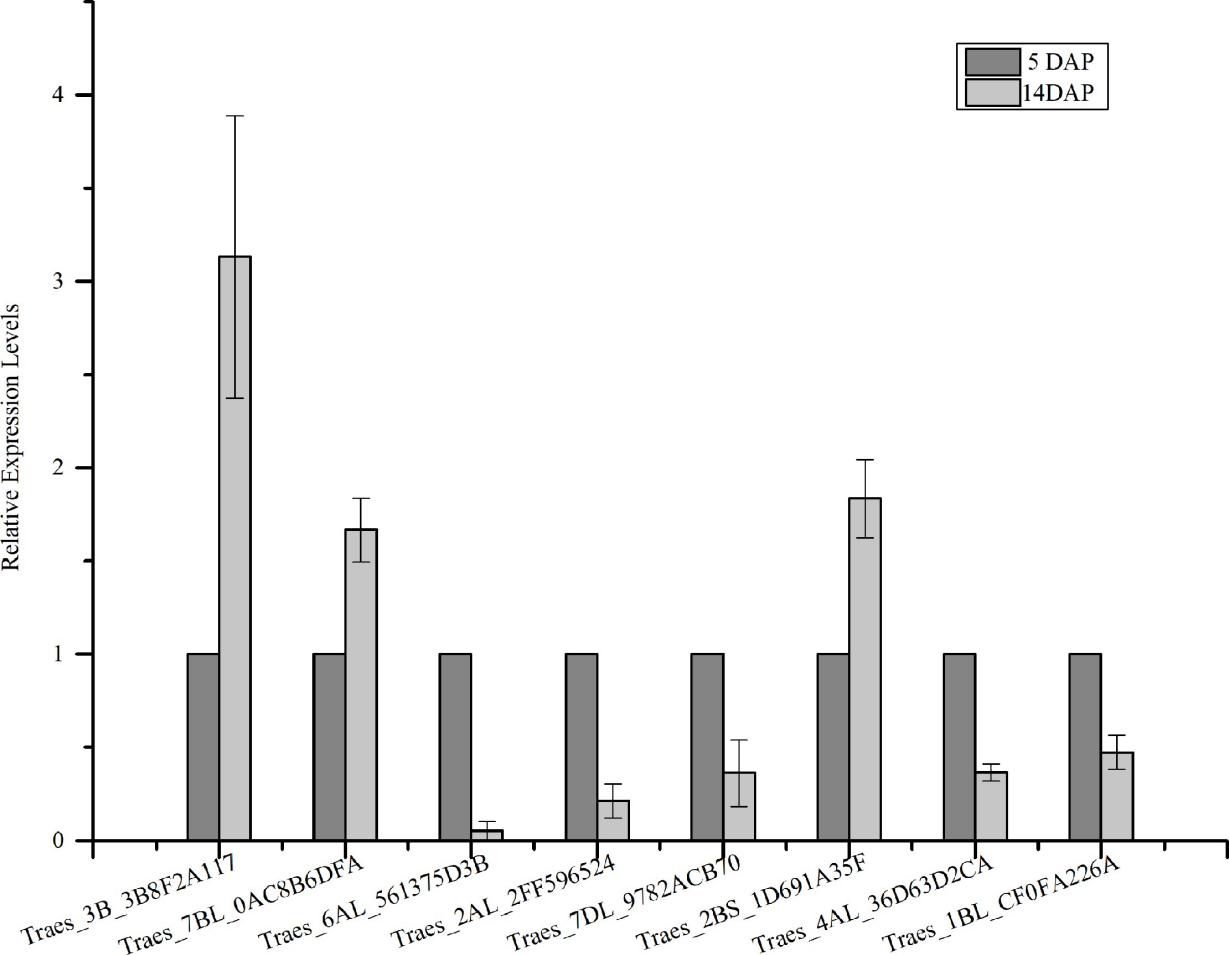
qRT-PCR analysis of 8 random selectively mRNA expression from transcriptome database. Values are the mean ± SD (standard deviation) of three independent experiments. T05, 5 DAP; T06, 14 DAP.

To confirm the results revealed by transcriptomic analysis, we carried out qRT-PCR to detect the expression of 8 randomly selected genes. The results showed that the dynamic expressions of selected genes were consistent with the transcriptome data, although the fold change had more or less difference (Fig 7). It suggested that our transcriptome data were reliable for all kinds of analysis.

## Discussion

In this study, we performed deep transcriptomic surveys of early wheat grains at two developmental stages (5 DAP and 14 DAP) to obtain dynamics of genes expression related to grain development. A total of 112,984 genes were generated, which provided abundant data for studying early grain development and 9,358 DEGs were found between 5 DAP and 14 DAP (S2 Table). Some candidate genes related to grain-size were identified, which were involved in carbohydrate metabolism, transcription, signal transduction, glycolysis/gluconeogenesis metabolism and protein processing in the endoplasmic reticulum. Our study showed that these metabolism pathways all participated in early wheat grain development. Through a comparative study of these two stages of grain development, we may predict the genes involved in grain enlargement and to some extent understand their regulating mechanisms.

Our results showed that most of the DEGs were located on B subgenome (Fig 2), and B subgenome may have a greater influence on early seed development. According to previous studies, genes associated with cell division, photosynthesis and development rather than storage product synthesis were expressed most highly during the earliest period [26]. GO analysis in our study showed that DEGs were involved in the cell part and cell (Fig 3), which is consistent with previous studies. KEGG analysis showed that DEGs were most enriched in plant hormone signal transduction, starch and sucrose metabolism and gluconeogenesis in our data. The expression pattern matched with the grain-size increase and development. According to previous studies, gibberellin and auxin are the main regulators of early grain development [27–28]. In our transcriptomic analysis, the expression levels of auxin-related genes significantly increased at 14 DAP, which is consistent with previous study.

Meanwhile, a lot of ethylene-responsive transcriptional factors (ERFs) are significantly regulated during early grain developmental stage. It was reported that ERFs could regulate cell and organ identity and promote cell proliferation and morphogenesis during embryo development. In addition, three jasmonate-related genes are also significantly regulated. As a result it was deduced that cross-talk of hormones might play an important role in early grain development.

There are lots of transcription factors detected in our data. Grain development is found to be controlled by some transcription factors in previous studies. For example, many WRKY family genes are reported to function under pathogen infection, stress and cold adaption [29], while several members of this family also play an important role in embryo development, germination and metabolic pathways [30]. Zinc-finger protein with transcription activation is reported to regulate vegetative development in plant and animal, and mutations of this family genes will lead to abnormal embryo and other morphological changes. The abundance changes of many WRKY family genes and Zinc-finger protein genes were found in our data (S3–S5 Tables). However, most of these genes were down-regulated. It indicated that these transcription factors might specifically function in early grain-size development other than initiation of grain filling and we need to validate further the functions of these genes in regulating grain-size development.

Starch is the main component of wheat grains and the main source of energy in human diets [31], so it is considered as a critical determinant of wheat yield and quality. However, the synthesis of starch in wheat grain is a complex process. Identifying genes involved in starch biosynthesis is of importance. In this study, most of the enzymes involved in starch biosynthesis were identified and they showed a high expression which was consistent with previous studies [32]. Glucose-1-phosphate adenylyltransferase (ADP-glucose pyrophosphorylase) is a major factor in grain starch biosynthesis. In this study, we found that most glucose-1-phosphate adenylyl transferases increased from 5 DAP to 14 DAP (S5 Table). It is shown that starch biosynthesis involves the transport of sucrose and its conversion to starch. In this study, DEGs involved in sucrose metabolism pathway was also enriched such as genes of sucrose synthase, sucrose-phosphate synthase and trehalose 6-phosphate synthase. Fructose-bisphosphate aldolase is a key factor in the gluconeogenesis pathway. In this study, expression levels of three fructose-bisphosphate aldolase genes were found to increase at 14DAP. Our data showed that glucose-6-phosphate isomerase apospory-associated protein and triosephosphate isomerase were also involved in gluconeogenesis and all the enzyme genes showed up-regulated at 14DAP. Sucrose is degraded by sucrose synthase and invertase toward starch production [33], and suppression of starch biosynthesis genes reduces grain starch which leads to a wrinkled phenotype [34]. Grain yield is mainly the consequence of starch accumulation. In all, these results indicated that the activity of sucrose and starch metabolism may be correlated with increasing grain sink strength and grain yield. It is helpful for increasing wheat yield to further inspect the function of these genes.

KEGG analysis showed that DEGs were also enriched in other important pathways in which early grain development is involved, such as phenylalanine metabolism, amino acid metabolism and protein processing in the endoplasmic reticulum (Fig 5), which is consistent with the results from *Arabidopsis* and rice [35–36]. A huge amount of secondary metabolites, such as flavonoid and lignin were derived from phenylalanine and tyrosine [37] and these secondary metabolites have many functions in plant development. In our data, 41 DEGs are related to secondary metabolism including genes of primary amine oxidase, aspartate aminotransferase, phenylalanine ammonia-lyase and nitrate reductase were significantly regulated indicating that phenylalanine may play an important role in early grain development. The protein content of wheat grains is a crucial determinant of quality and economic factors [38]. Therefore, protein synthesis is very important for wheat grain development. The final grain protein content (GPC) has been correlated with the free amino acid concentrations and the expression of genes involved in amino acid metabolism significantly changed (Fig 5, S2 Table), as a result it can be deduced that the expression of these genes at 14 DAP may have an effect on final GPC and initiated grain filling. Nitrate reductase activity was highly correlated to N absorption and protein content and our data showed that the expression of nitrate reductase significantly increased. In addition, mutation of genes related to primary metabolism can strangely perturb early grain development [39], and little is known on the mechanisms of grain development through the primary metabolism. Therefore, our focus will be on constructing metabolic networks and characterizing genes related to primary metabolism in the future. In all, our study provided genome-wide gene expression profiles during early grain development and identified some important candidate genes in wheat. We produce a valuable resource for analyzing temporal and spatial expression of genes and further understanding their molecular and cellular functions during early grain development in wheat.

## Conclusions

This study performed transcriptome analyses of immature wheat grains at 5 DAP and 14 DAP during grain enlargement stage, and 12,555 new genes and 9,358 differentially expressed genes (DEGs) were obtained. The important candidate genes related to grain-size have been identified and they were involved in carbohydrate metabolism, transcription, signal transduction, glycolysis/gluconeogenesis metabolism and protein processing in the endoplasmic reticulum. Most of the DEGs were located on B subgenome, especially chromosome 3B. B subgenome may have a greater influence on grain enlargement. KEGG analysis showed that DEGs were most enriched in starch and sucrose metabolism, plant hormone signal transduction and gluconeogenesis. Forty-one DEGs related to secondary metabolism were significantly regulated, indicating that they may likely play an important role in early grain development. The expression of genes involved in amino acid metabolism significantly changed, we deduce the expression of these genes at 14 DAP may have an effect on final GPC, and they are likely to initiate grain filling together with those DEGs related to starch and sucrose metabolism. Further function analysis of important candidate genes related to grain-size will provide crucial clues to reveal the mechanisms underlying grain pattern formation in common wheat.

## Materials and methods

### Plant materials

“Bainong 4199” a major cultivar in China, has the characteristics of full grain and high yield. It was planted and grown under non-stressed conditions from October 2015 to June 2016 in the field at the experimental farm of Henan Institute of Science and Technology, Xinxiang, China. In April 2016, immature grains of “Bainong 4199” were collected at 5 DAP and 14 DAP and was immediately submerged into liquid nitrogen for use. Respective mixtures of immature grains at 5 DAP and 14 DAP were used as samples. According to previous report, 14 DAP is the transition stage from grain enlargement to grain filling [40–41], so we collected 5 DAP and 14 DAP grains to study gene expression profile of wheat grain at enlargement stage.

### RNA extraction, cDNA library construction and sequencing

Total RNA was isolated from grains of 5 DAP and 14 DAP using TRIzol reagent (Invitrogen, Carlsbad, CA, USA) according to the manufacturer’s instructions. RNase-free DNase was used in removing the residual DNA for 30 min at 37°C. The extracted RNA was qualified and quantified using a NanoDrop 2000 UV–Vis spectrophotometer (NanoDrop, Wilmington, DE, USA) and the samples showed a 260/280 nm ratio between 1.8 and 2.2 and an OD260/230 > 1.0. For each of the samples at 5 DAP and 14 DAP, three replicates of each time point were performed for the RNA-seq.

The mRNA-seq libraries were prepared using the Illumina TruSeq RNA Sample Preparation Kit (Illumina Inc., San Diego, CA, USA) and the libraries were constructed for sequencing using Illumina HiSeqTM 2500 sequencing platform (Illumina Inc., San Diego, CA, USA) at Biomarker Technologies Corporation in Beijing. Clean data (clean reads) were obtained by removing reads containing adapter, empty reads and low quality reads from raw data. Real-time monitoring was performed for each cycle during sequencing and the ratio of high-quality reads with quality scores greater than Q30 for the raw reads and guanine-cytosine (GC) content was calculated for quality control.

### Differential expression analysis and differentially expressed genes (DEGs)

Tophat (v2.0.12) is a fast mapping tool for RNA-seq reads; it can identify splice junctions between exons [42]. Cufflinks can be used to assemble the reads into transcripts based on the mapping results [43]. Clean reads were aligned to the reference genome (www.ncbi.nlm.nih.gov/dbEST/dbEST_summary.html) using Tophat and Cufflinks. Per Kilobase of exon per Million fragments mapped (FPKM) method is used as a method for estimating gene expression levels. Differential expression analysis of two samples was performed using the DESeq fit for duplicate biological samples [44]. To designate changes in gene expression, FDR of 0.001 and log2 (Fold change) of 1.5 were set as the threshold for differential expression.

### Functional annotation

Gene ontology (GO) enrichment of differentially expressed genes was implemented to analyze gene function. The Cluster of Orthologous Groups of proteins (COG) database is based on the phylogenetic relationships among bacteria, algae and eukaryotes. Genes in an orthologous relationship can be classified using the COG database [45]. To understand functions and utilities of DEGs, we used KOBAS software to test the statistical enrichment of DEGs in KEGG (InterPro scan and the Kyoto Encyclopedia of Genes and Genomes) pathways [46]. In this study, we analyzed functions of new genes and DEGs by performing a blast search against the COG, GO, KEGG pathway, Swiss-Prot and Non-redundant protein (NR) databases.

### Gene expression level validation by qRT-PCR

Total RNA was extracted by TransZol Plant (Transgen) and it was reverse-transcribed with PrimerScript^TM^ RT regent Kit with gDNA Eraser (Takara) according to the manufacturer’s instruction. A 5 μl aliquot of 1:50 diluted cDNA was used as the template in a 10 μl PCR system. The qRT-PCR was performed using TransStar Green qPCR Supermix (Transgen) after a pre-incubation at 95°C for 30s, followed by 40 cycles of denaturation at 95°C for 5 s, annealing and extension at 60°C for 30 s in an Applied Biosystems 7500 Fast Real-Time PCR Systems. The gene-specific primers of qPCR were listed in S6 Table and wheat actin was used as an internal standard to normalize the gene expression. The quantification of gene expression was determined in three independent samples.

## Supporting information

S1 Fig KEGG pathway analysis identified the plant hormone signal transduction (ko04075) for DEGs between 5 DAP and 14DAP. Red, green and blue indicate genes expression increase, decrease and mixed change at 14 DAP respectively.
(PPTX)

S2 Fig KEGG pathway analysis identified starch and sucrose metabolism (ko00500) for DEGs between 5 DAP and 14DAP. Red, green and blue indicate genes expression increase, decrease and mixed change at 14 DAP respectively.
(PPTX)

S3 Fig KEGG pathway analysis identified phenylalanine metabolism (ko00360) for DEGs between 5 DAP and 14DAP. Red, green and blue indicate genes expression increase, decrease and mixed change at 14 DAP respectively.
(PPTX)

S1 Table The list of new genes and sequences. (XLSX)

S2 Table Total DEGs between 5 DAP and 14 DAP. (XLSX)

S3 Table The GO classification of DEGs. (XLSX)

S4 Table The COG classification of DEGs. (XLSX)

S5 Table KEGG pathway annotation of DEGs. (XLSX)

S6 Table Primers for quantitative real-time PCR. (XLSX)
