# Supplementary material for: Transcriptome analysis reveals important candidate genes involved in grain-size formation at the stage of grain enlargement in common wheat cultivar “Bainong 4199”
Source: PLoS One. 2019 Mar 25;14(3):e0214149. doi: 10.1371/journal.pone.0214149 (PMC6433227; doi:10.1371/journal.pone.0214149)
Supplement: S3 Fig — Red, green and blue indicate genes expression increase, decrease and mixed change at 14 DAP respectively. (PPTX) [file pone.0214149.s003.pptx]

## Slide 1
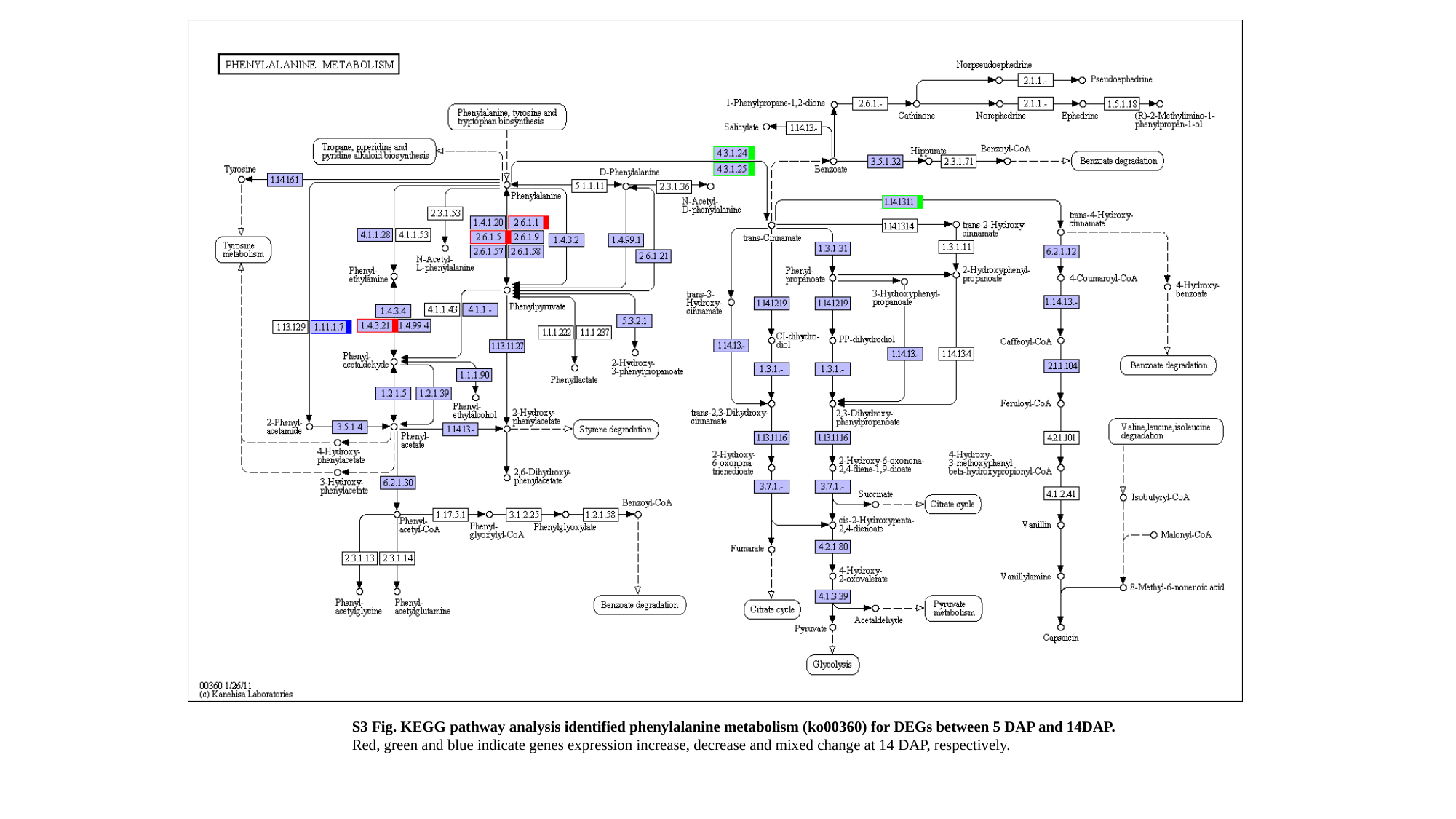

S3 Fig. KEGG pathway analysis identified phenylalanine metabolism (ko00360) for DEGs between 5 DAP and 14DAP. Red, green and blue indicate genes expression increase, decrease and mixed change at 14 DAP, respectively.
